# Measurement properties of instruments to assess insight in psychosis: A systematic review protocol

**DOI:** 10.1371/journal.pone.0316447

**Published:** 2025-01-24

**Authors:** Hadar Hazan, Melissa C. Funaro, Vinod H. Srihari

**Affiliations:** 1 Department of Psychiatry, Yale University School of Medicine, New Haven, Connecticut, United States of America; 2 Program for Specialized Treatment Early in Psychosis (STEP), Connecticut Mental Health, New Haven, Connecticut, United States of America; 3 Yale University Harvey Cushing/John Hay Whitney Medical Library, New Haven, Connecticut, United States of America; Northwestern University Feinberg School of Medicine, UNITED STATES OF AMERICA

## Abstract

**Introduction:**

Insight in psychosis, defined as a patient’s awareness and judgment of their mental illness, is a complex and evolving concept. Historically, the absence of insight was considered a defining characteristic of psychosis, but recent decades have seen the development of structured tools for its assessment. This systematic review aims to critically appraise the measurement properties of instruments used to assess insight in individuals with schizophrenia spectrum, bridging the gap between theoretical conceptualization and clinical practice.

**Methods:**

This protocol is reported following the Preferred Reporting Items for Systematic Review and Meta-Analysis Protocols (PRISMA-P) guidelines. Eligible studies will evaluate instruments measuring insight in psychosis, including self-report, observer ratings, behavioral measurements, and semi-structured interviews. The psychometric properties assessed will encompass reliability, validity, responsiveness, interpretability, and clinical utility. We will include primary quantitative studies published in peer-reviewed journals and available in English.

**Results:**

Two reviewers will conduct data extraction and quality assessment independently, with discrepancies resolved through consensus or a third reviewer. The COSMIN Risk of Bias checklist and the GRADE approach will be used to evaluate each measure’s methodological quality and overall strength of evidence.

**Discussion:**

This systematic review will synthesize current evidence on insight measurement in psychosis, providing a comprehensive overview of the strengths and limitations of existing instruments. The findings will inform future research and clinical practice, facilitating the selection of the most appropriate tools for assessing insight and ultimately contributing to improved care for individuals with psychosis. The findings will provide clinicians and researchers with robust tools for assessing insight, ultimately enhancing treatment outcomes for individuals with psychosis.

## Introduction

Insight in psychosis, broadly defined as a patient’s awareness and judgment of their mental illness, has been a contentious and evolving concept throughout psychiatric history. Before the mid-19th century, the absence of insight was often considered a defining characteristic of psychosis. The concept of *insight in psychosis* seemed contradictory, as insanity was primarily understood as the manifestation of delusional cognition. However, the emergence of concepts like *self*, *consciousness*, and *introspection* in the mid-19th century, along with the inclusion of subjective experiences in the definition of insanity, gradually paved the way for comprehending the possibility of individuals recognizing their mental illness [1].

Scholars have since attempted to conceptualize insight in psychosis while acknowledging its elusive nature. In his 1934 essay “The Psychopathology of Insight,” Aubrey Lewis [2] described insight as the “correct attitude to a morbid change in oneself,” while noting that this definition is not final and that each of the terms—correct, attitude, morbid, change in oneself—calls for discussion. A major discussion did not immediately follow, and the systematic investigation of insight remained relatively neglected throughout much of the 20th century. For several decades following Lewis’s work, the assessment of insight relied primarily on impressionistic and anecdotal methods rather than systematic evaluation [3–7].

In 1989, McEvoy et al. [8] introduced the first structured insight scale, marking a significant advancement. Since then, various insight measures have emerged, encompassing cognitive, attitudinal, symptom-specific, and temporal aspects. These measures vary in their assessment approaches, ranging from binary to spectrum-based evaluations, and in perspective, whether self-assessed or clinician-rated.

Despite these advancements, a persistent gap remains between the theoretical conceptualization of insight and its clinical manifestation [9]. The concept of insight, as tentatively defined by Lewis, comprises both *awareness* of a change in experience and a *judgment* of what the change means and how it impacts one’s functioning and interaction with the world [10]. This complexity poses significant challenges for researchers and clinicians alike, with different scales focusing on different aspects of insight.

The varied use of the concept of insight highlights a lack of consensus on its definition and measurement. For instance, the RAISE study found that the Coordinated Specialty Care (CSC) program significantly improved insight compared to community care over two years, as measured by a single item from the PANSS, which conflates insight’s judgment and awareness components. In contrast, McEvoy et al. [11], found no improvement in insight among involuntarily committed schizophrenia patients, using the Insight and Treatment Attitudes Questionnaire (ITAQ), which assesses patients’ awareness of their mental disorder and the need for treatment, capturing both cognitive recognition and attitudinal acceptance. David et al. [12] reported significant fluctuations in insight among schizophrenia patients over time, measured by the Schedule for Assessment of Insight (SAI), which evaluates awareness of illness, relabeling of symptoms, and treatment compliance. These variations in measurement tools complicate comparisons across studies [13–15].

Given the critical role of insight in understanding and treating psychosis, there is a need for a comprehensive evaluation of existing insight measures. This systematic review aims to address the challenges in assessing insight in individuals diagnosed with psychosis by critically appraising the various measures employed. By doing so, we seek to provide clarity on the most psychometrically valid and clinically useful tools available, facilitating more consistent and comparable research in the future.

This review will focus on answering the following question: What are the psychometric properties of measures that have been employed to assess insight in individuals diagnosed with psychosis? By conducting this systematic review, we aim to provide researchers and clinicians with a comprehensive understanding of the strengths and limitations of existing insight measures. This knowledge will inform the selection of appropriate tools for future studies and clinical assessments, ultimately contributing to more robust and comparable research in the field of psychosis.

## Methods

The Preferred Reporting Items for Systematic Review and Meta-Analysis Protocols (PRISMA-P) statement has been followed in the reporting of this systematic review protocol [16]. The completed PRISMA-P checklist can be found in S1 Table.

### Eligibility criteria

To be included in this review, studies need to meet the subjoined inclusion criteria. These are defined according to the COSMIN criteria:

### Construct of interest

Studies that evaluate instruments that aim to measure the construct of insight in psychosis. Awareness of illness is also considered when related to insight in psychosis.

### Population of interest

Studies that encompass individuals diagnosed with schizophrenia spectrum disorder.

### Type of measurement instrument

Studies that investigate a relevant measurement instrument including self-report, observer ratings, behavioral measurement, multidimensional measurement, and semi-structured Interviews.

### Measurement properties

For psychometric properties questions, relevant outcomes are reliability (including internal consistency, test-retest, and inter-rater reliability), validity (content, construct, and criterion), responsiveness, interpretability, and clinical utility, as defined by COSMIN. Each included measure will be evaluated on these properties using the COSMIN risk of bias checklist. Studies reporting on any of these properties will be included, with a note on which properties are assessed for each measure.

### Eligible study types

Only primary, quantitative studies will be included in this review. Each study must have a minimum sample size of ten participants. Studies should explicitly state their aim to evaluate the use of an existing measurement instrument or to develop a new one, following COSMIN guidelines [17]. To ensure quality and accessibility, only studies published in peer-reviewed journals and available in full text in English will be considered.

### Literature searches

An experienced medical librarian (MCF) will be consulted on methodology and a medical subject heading (MeSH) analysis of known articles provided by the research team will be done using the Yale MeSH Analyzer [mesh.med.yale.edu/]. Scoping searches will be performed in each database, and an iterative process will be used to translate and refine the searches.

To maximize sensitivity, the formal search will use controlled vocabulary terms and synonymous keywords to capture the concepts of "psychosis", "insight", and "measurements". Results will be limited to publications in English, but no date limits will be imposed on the search. This approach will ensure a comprehensive review of the literature, including early conceptualizations of insight in psychosis.

The search strategy will be peer-reviewed by a second librarian not otherwise associated with the project, enhancing the rigor of our methodology. To ensure comprehensive coverage, reviewers will check for additional relevant citing articles using included studies. To capture recently published articles, a second database search will be rerun before publishing the paper, ensuring the inclusion of the most up-to-date research.

The search consists of five search strategies combined with the ‘AND’ Boolean operator, focusing on:

1. **Disorders**: ‘Schizophrenia Spectrum and Other Psychotic disorders’, ‘psychosis’, ‘psychoses’, ‘psychotic’, and ‘schizo*’
2. **Assessment**: ‘assessment*’, ‘checklist*’, ‘Interview Psychological’, ‘instrument*’, ‘inventor*’, ‘measure*’, ‘questionnaire*’, ‘scale*’, ‘score*’, ‘survey*’, ‘test’, ‘tests’, ‘tool*’, ‘Surveys and Questionnaires’
3. **Insight**: ‘insight*’ and ‘awareness*’
4. **Illness Awareness**: ‘illness awareness*’
5. **Illness Attitudes/Beliefs**: ‘illness attitudes*’ and ‘illness beliefs*’

The searches will be conducted in five electronic databases. These databases include Ovid MEDLINE ALL, APA PsycInfo (Ovid), Embase (Ovid), Web of Science Core Collection (Clarivate), and Health and Psychosocial Instruments Database (HaPI) (Ovid). Each database will be systematically searched to identify pertinent research articles, ensuring a broad collection of literature relevant to the topic. As an example, the full search strategy that will be used in Ovid MEDLINE ALL can be found in Table 1.

**Table 1:** Comprehensive search strategy for Ovid MEDLINE ALL.

Step	Concept	Search Terms
1	Psychosis and Schizophrenia	exp psychotic disorders/ or exp Schizophrenia/
1	Insight and Awareness	*Awareness/ or *Attitude/ or *"Attitude to Health"/
1	Assessment Tools	exp "Surveys and Questionnaires"/ or exp Interview, Psychological/ or exp Psychiatric Status Rating Scales/
2	Psychosis and Insight (Keywords)	((psychosis or psychoses or psychotic or schiz*) adj7 (insight* or awareness or attitude* or belief* or perception or self-awareness or self-understanding or cognizan* or realization or intuition or discernment or grasp or perceptive or perceptiveness or savvy or unawareness or comprehension)).tw,kw.
3	Assessment Tools (Expanded)	exp "Surveys and Questionnaires"/ or exp Interview, Psychological/ or exp Psychiatric Status Rating Scales/ or (assessment* or checklist* or check list* or instrument* or inventor* or measure* or questionnaire* or scale or scales or score or scores or survey or surveys or test or tests or tool or tools or interview*).tw,kw.
4	Psychometric Properties	"Reproducibility of Results"/ or Psychometrics/ or (reliability or reliabl* or validate$ or validation or validity or psychometric* or responsiv* or interpretab* or "measurement error*" or "error of measurement*" or "errors of measurement*" or consistency or "factor analys*" or "factor structure" or "item analys*" or "item response" or "item functioning" or "component analys*" or "measurement invariance" or "test construction" or sensitivity or specificity or "statistical rotation" or structured or semistructured or "oblique rotation" or "orthogonal rotation" or "varimax rotation" or "split half" or "test retest" or interrater or "inter rater" or intrarater or "intra rater" or stability or equivalence or "intraclass correlation coefficient*" or "intra-class correlation coefficient*" or kappa or "coefficient of correlation" or "coefficients of correlation" or "correlation coefficient*" or "pearson product moment" or "spearman rho" or "minimal detectable difference" or "minimal detectable change").tw,kw.
5	Combination	2 and 3 and 4
6	Combination	1 or 5
7	Language Limit	limit 6 to English language

All identified citations will be imported into EndNote 21 and deduplicated using the Yale Reference Deduplicator tool [library.medicine.yale.edu/reference-deduplicator]. The deduplicated list will then be imported into Covidence [covidence.org] for the screening process.

### Study selection

**Fig 1:**
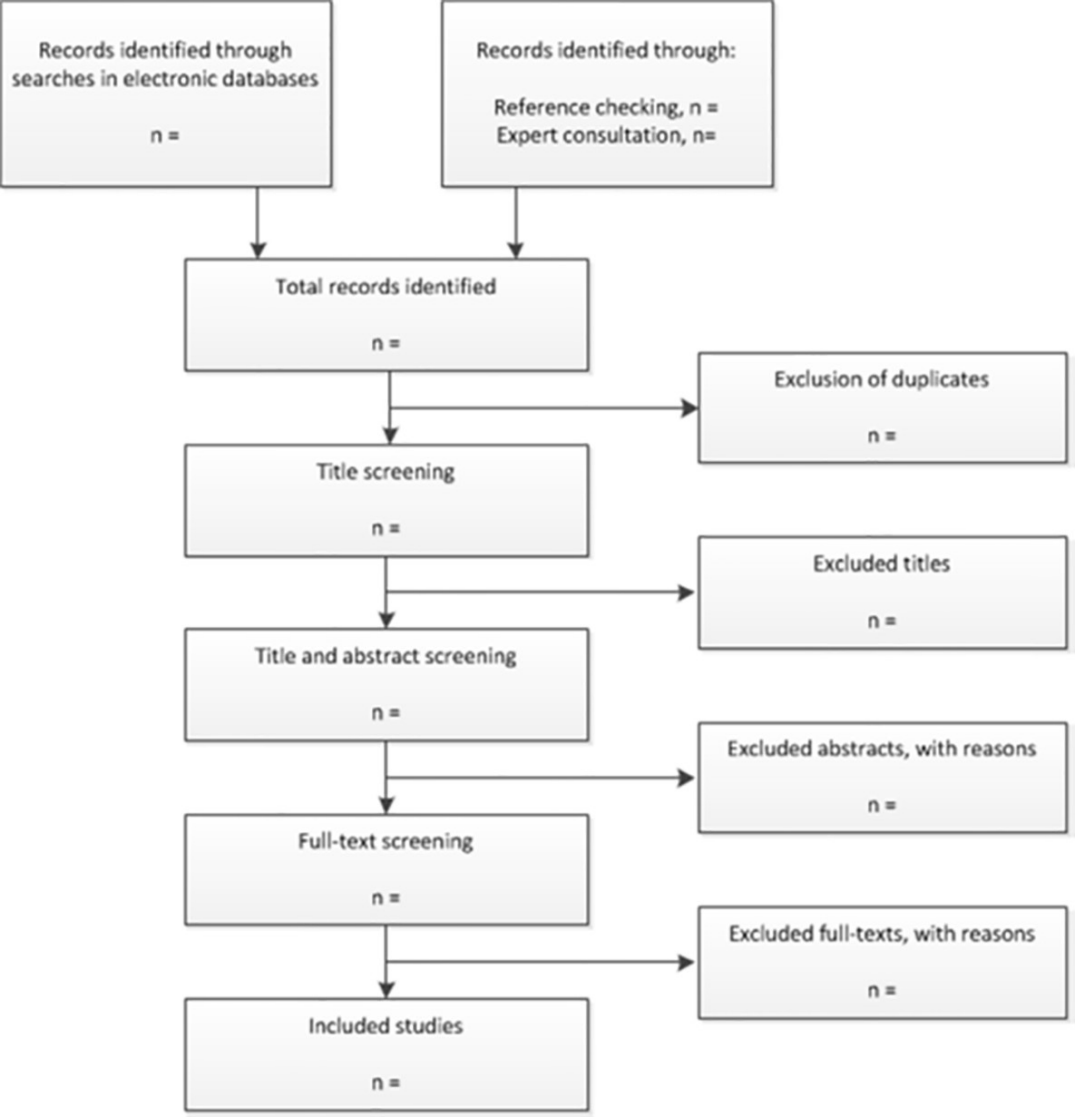
Flow diagram of the study selection process.

Following the initial search (stage 1), two more stages will follow (Fig 1).

### Title and abstract selection

The second stage will consist of selection based on title and abstract. The entire selection will be performed in duplicate by two independent reviewers (1: HH, 2: ST) A pilot screening will be conducted with the first 100 citations to ensure consistency in the application of inclusion/exclusion criteria. Following the pilot, discrepancies will be resolved by consensus between the two reviewers. If consensus cannot be reached, a third reviewer ([VS]) will be consulted for arbitration.

### Full-text selection

In the third stage, the same two independent reviewers will perform the selection of the full texts. Discrepancies will be resolved by consensus between the reviewers. If consensus cannot be reached, the third reviewer will be consulted for a final decision.

### Data extraction

Two reviewers will perform Data extraction independently using a standardized form designed specifically for this systematic review on insight measures in psychosis. The form will capture: (1) general study information (e.g., first author, title, journal, year, funding source), (2) study design characteristics (e.g., study design, setting, duration, sample size), (3) participant characteristics (e.g., age, gender, diagnosis, age at diagnosis, duration of illness, symptom severity), (4) insight measure details (e.g., name of the measure, type of measure, number of items, scoring method, dimensions of insight assessed), (5) psychometric properties (e.g., reliability, validity, responsiveness, interpretability), and (6) associate outcome variables (e.g., quality of life, treatment adherence).

The form will be pilot tested on a representative sample of studies and optimized if necessary. Reviewers will discuss completed forms, resolving discrepancies by consensus or, if needed, consultation with a third reviewer. In cases of missing information, study authors will be contacted. This comprehensive approach ensures thorough and accurate data collection, capturing not only the basic study and participant information but also detailed aspects of the insight measures and their psychometric properties, which are crucial for evaluating their validity and utility in assessing insight in individuals with psychosis.

### Quality assessment

Two reviewers will independently assess the methodological quality of the included studies using the COSMIN Risk of Bias checklist for Patient-Reported Outcome Measures (PROMs). This checklist is specifically designed for evaluating the quality of studies on measurement properties and will be adapted as necessary for clinician-rated measures. The COSMIN checklist covers various measurement properties including reliability, validity, responsiveness, and interpretability. For each study, reviewers will evaluate the methodological quality of the reported measurement properties. The assessment will consider whether the statistical methods are based on Classical Test Theory (CTT) or Item Response Theory (IRT), with appropriate standards applied for each. The COSMIN checklist uses a 4-point rating scale: "very good," "adequate," "doubtful," and "inadequate." This rating will be applied to each measurement property reported in each study. Discrepancies between reviewers will be resolved through discussion or, if necessary, consultation with a third reviewer.

In addition to the COSMIN checklist, reviewers will assess the risk of bias in individual studies, considering factors such as sample size, participant selection, and handling of missing data. They will also evaluate the overall quality of evidence for each measurement property across studies using the GRADE approach adapted for measurement properties studies.

Regarding publication bias, the reviewers will qualitatively assess potential bias by looking for patterns in the reported results, such as a preponderance of studies reporting only positive findings. They will also consider the comprehensiveness of the search strategy and any indications of selective outcome reporting within studies.

The reviewers will also assess the generalizability of the findings, considering factors such as the characteristics of the study populations, the contexts in which the insight measures were used, and any limitations reported by the study authors. This comprehensive quality assessment will help identify the insight measures with the strongest psychometric properties and provide a clear understanding of the quality and potential biases in the existing evidence base for insight measurement in psychosis.

## Synthesis of results

We will prepare a comprehensive table of characteristics for all included studies, detailing information on study design, sample characteristics, insight measurement instrument(s), and reported outcomes. This table will provide a clear overview of the existing literature on insight measures in psychosis.

Outcome tables will be prepared for each insight measurement instrument (e.g., the Scale to Assess Unawareness of Mental Disorder, the Beck Cognitive Insight Scale) and each outcome construct (e.g., clinical insight, cognitive insight) separately. These tables will summarize the psychometric properties reported for each measure, including reliability, validity, responsiveness, and interpretability.

We will also create a table categorizing insight measures according to their primary focus or dimension of insight assessed, such as awareness of illness, attribution of symptoms, need for treatment, cognitive insight (self-reflectiveness and self-certainty), and multidimensional measures.

To provide a comprehensive overview, we will develop a quality matrix. This matrix will list all included insight measurement instruments and summarize key information including the type of measure (self-report, clinician-rated, or multi-informant), number of studies using the measure, population characteristics (e.g., diagnosis, age range, illness duration), COSMIN quality scores for each psychometric property (using the 4-point rating scale), and overall strength of evidence (based on GRADE approach). This quality matrix will allow readers to quickly identify the most appropriate insight measurement instruments with the strongest psychometric properties for different contexts and populations within psychosis research.

We will also present a narrative synthesis discussing our findings. This synthesis will summarize the overall state of insight measurement in psychosis, discuss strengths and limitations of existing measures, identify gaps in the current evidence base, provide recommendations for the use of insight measures in different research and clinical contexts, and suggest directions for future research in insight measurement.

This comprehensive approach to data synthesis and presentation will provide a clear, accessible, and informative overview of the current state of insight measurement in psychosis, facilitating evidence-based selection of measures for future research and clinical practice.

## Discussion

In this protocol, we have outlined our approach for a systematic review of the measurement properties of instruments used to assess insight in individuals with psychosis. This comprehensive overview is crucial for several reasons. Insight in psychosis is a complex and multidimensional construct that has been conceptualized and measured in various ways over the years. The evolution of our understanding of insight necessitates a thorough evaluation of existing measurement tools to ensure they accurately capture the current conceptualization of insight in psychosis.

There is significant heterogeneity in the presentation of psychosis across individuals, which may influence the manifestation and assessment of insight. Factors such as illness duration, symptom severity, and cognitive functioning can all impact an individual’s level of insight, underscoring the need for measures that can capture this variability.

Insight in psychosis has important clinical implications. It has been associated with treatment adherence, clinical outcomes, and functional recovery. Therefore, having psychometrically sound measurement instruments is crucial for both research and clinical practice. These tools need to be reliable, valid, and sensitive to change to accurately assess insight and monitor its fluctuations over time or in response to interventions.

The assessment of insight often relies on both self-report and clinician-rated measures. Each approach has its strengths and limitations, and a comprehensive review of available tools is necessary to guide researchers and clinicians in selecting the most appropriate measures for their specific needs and contexts.

Currently, there is growing interest in personalized approaches to the treatment of psychosis, with insight being recognized as a potential target for intervention. Accurate measurement of insight is the critical first step in determining the need for such interventions and evaluating their effectiveness. When insight is not adequately assessed, opportunities for targeted interventions may be missed, potentially impacting patient outcomes.

To address these needs, we have initiated this systematic review aimed at improving the assessment and understanding of insight in psychosis. This review of the literature describing insight measurement instruments is a crucial step toward formulating evidence-based recommendations for both research and clinical practice.

By providing a comprehensive overview of available insight measures, their psychometric properties, and their applicability in different contexts, we aim to facilitate more informed decision-making in the selection and use of these tools. This, in turn, will contribute to more robust research in the field and potentially lead to improved clinical assessment and care for individuals with psychosis.

In conclusion, this systematic review will fill an important gap in the literature by synthesizing the current evidence on insight measurement in psychosis. The findings will not only inform future research but also have practical implications for clinical assessment and intervention planning in the care of individuals with psychosis.

## Supporting information

S1 Table PRISMA-P 2015 checklist—Measurement properties of instruments to assess insight in psychosis: A systematic review protocol. (DOCX)
